# Low-Intensity Ultrasound-Induced Anti-inflammatory Effects Are Mediated by Several New Mechanisms Including Gene Induction, Immunosuppressor Cell Promotion, and Enhancement of Exosome Biogenesis and Docking

**DOI:** 10.3389/fphys.2017.00818

**Published:** 2017-10-23

**Authors:** Qian Yang, Gayani K. Nanayakkara, Charles Drummer, Yu Sun, Candice Johnson, Ramon Cueto, Hangfei Fu, Ying Shao, Luqiao Wang, William Y. Yang, Peng Tang, Li-Wen Liu, Shuping Ge, Xiao-Dong Zhou, Mohsin Khan, Hong Wang, Xiaofeng Yang

**Affiliations:** ^1^Department of Ultrasound, Xijing Hospital and Fourth Military Medical University, Xi'an, China; ^2^Departments of Pharmacology, Microbiology and Immunology, Centers for Metabolic Disease Research, Cardiovascular Research, and Thrombosis Research, Lewis Katz School of Medicine at Temple University, Philadelphia, PA, United States; ^3^Department of Cardiovascular Medicine, First Affiliated Hospital of Kunming Medical University, Kunming, China; ^4^Department of Orthopedics, Beijing Charity Hospital of China Rehabilitation Research Center, Beijing, China; ^5^Heart Center, St. Christopher's Hospital for Children, Drexel University College of Medicine, Philadelphia, PA, United States; ^6^Deborah Heart and Lung Center, Browns Mills, NJ, United States

**Keywords:** ultrasound, anti-inflammatory gene induction, exosomes, immunosuppressor cells, ultrasound for cancer therapy

## Abstract

**Background:** Low-intensity ultrasound (LIUS) was shown to be beneficial in mitigating inflammation and facilitating tissue repair in various pathologies. Determination of the molecular mechanisms underlying the anti-inflammatory effects of LIUS allows to optimize this technique as a therapy for the treatment of malignancies and aseptic inflammatory disorders.

**Methods:** We conducted cutting-edge database mining approaches to determine the anti-inflammatory mechanisms exerted by LIUS.

**Results:** Our data revealed following interesting findings: (1) LIUS anti-inflammatory effects are mediated by upregulating anti-inflammatory gene expression; (2) LIUS induces the upregulation of the markers and master regulators of immunosuppressor cells including MDSCs (myeloid-derived suppressor cells), MSCs (mesenchymal stem cells), B1-B cells and Treg (regulatory T cells); (3) LIUS not only can be used as a therapeutic approach to deliver drugs packed in various structures such as nanobeads, nanospheres, polymer microspheres, and lipidosomes, but also can make use of natural membrane vesicles as small as exosomes derived from immunosuppressor cells as a novel mechanism to fulfill its anti-inflammatory effects; (4) LIUS upregulates the expression of extracellular vesicle/exosome biogenesis mediators and docking mediators; (5) Exosome-carried anti-inflammatory cytokines and anti-inflammatory microRNAs inhibit inflammation of target cells via multiple shared and specific pathways, suggesting exosome-mediated anti-inflammatory effect of LIUS feasible; and (6) LIUS-mediated physical effects on tissues may activate specific cellular sensors that activate downstream transcription factors and signaling pathways.

**Conclusions:** Our results have provided novel insights into the mechanisms underlying anti-inflammatory effects of LIUS, and have provided guidance for the development of future novel therapeutic LIUS for cancers, inflammatory disorders, tissue regeneration and tissue repair.

## Introduction

Ultrasound, alone or combined with contrast agent microbubbles have wide spectrum of applications ranging from well-established diagnostic tools (Wang et al., 2010; de Castro et al., 2012) to drug delivery (Schroeder et al., 2009; Tuckett et al., 2017), and other therapeutic methods. Ultrasound therapy now is widely used in clinical practice and clinical/translational research in the treatment of various human malignancies and pathologies including breast cancer, leukemia, lymphoma, melanoma, pancreatic neuroendocrine tumors (Kulke et al., 2011), hepatic cancer, nasopharyngeal cancers, glioma, ovarian cancer, colon cancer, gastric cancer (Wang et al., 2010) also in murine sarcoma (Copelan et al., 2015; Wood and Sehgal, 2015; McHale et al., 2016), stroke (Mijajlovic et al., 2013), prostatic hyperplasia, renal masses (Roberts, 2014), treatment of abdominal subcutaneous adipose tissue (Friedmann, 2015), bone repair (Padilla et al., 2014), osteoarthritis (Rutjes et al., 2010), and carpal tunnel syndrome, etc. (Page et al., 2012).

So far, several therapeutic ultrasound formats have been developed including high intensity focused ultrasound (Copelan et al., 2015) and low-intensity pulsed ultrasound (Sato et al., 2015). Recently, several clinical trials and experimental data verified the ability of ultrasound to elicit anti-inflammatory and tissue repair/regeneration responses (Johns, 2002; Mele et al., 2016) that led to identification and development of ultrasound as a novel therapeutic method (ElHag et al., 1985; Hashish et al., 1986; Chung et al., 2012; Kravchenko et al., 2013; Nagao et al., 2017). Nevertheless, the molecular and cellular mechanisms that exert the anti-inflammatory and immunosuppressve effects of LIUS remain poorly determined. Several reports have demonstrated that LIUS affects various immune cells and other cells involved in inflammatory regulation. For example, ultrasound has been shown to promote vasodilation, enhance blood flow, promote fibroblast and osteoblast proliferation and increase other cellular components leading to wound healing etc. (Johns, 2002).

Ultrasound can be performed without any contrasting agents. However, gas filled microbubbles as intravascular contrasting agents has been used for decades and was shown to be beneficial when imaging in many clinical scenarios (Kiessling et al., 2012). The application of microbubbles and ultrasound to deliver nanoparticle carriers for drug and gene delivery is a research area that has expanded greatly in recent years. The ability of ultrasound together with microbubbles to enhance drug delivery to a focused tissue had been attributed to many properties including sonoporation and microbubble mediated cavitation. Recent studies reported that utilization of ultrasound contrast microbubbles causes the so-called “sonoporation” effect (Sheikov et al., 2004; Forbes and O'Brien, 2012), which has been recognized as a significant factor in transient disruption of cell membrane permeability (Kravchenko et al., 2013) that allows easier transport of extracellular compounds into the cytoplasm of viable cells (Izadifar et al., 2017). Ultrasound cavitation is defined as the formation or activity of gas-filled bubbles in medium exposed to ultrasound. The pressure wave passing through the medium can make these gas bubbles to oscillate, which creates a circulating fluid flow around the bubble where the velocity and sheer stress of the flow depend on the oscillation amplitude. The sheer stress created by cavitation can disrupt the surrounding vesicles, including drug carrying nanoparticles or micelles and release its contents. Many nanoparticle delivery vehicles show promise for carrying high therapeutic payloads, controllable release rates, and targeting abilities—both passive and active. Also physical effects of cavitation may disrupt cell membranes and increase cellular and micro-vascular permeability that lead to enhanced drug uptake (Husseini and Pitt, 2008; Danhier et al., 2010; Kruse et al., 2010). Despite many studies published explaining the enhanced drug delivery of LIUS technology, molecular and cellular mechanisms underlying its anti-inflammatory effects remain unknown.

Exosomes are endosomal-derived nanoscale vesicles that are released by most cell types and are present in all eukaryotic fluids, including blood, urine, and cultured medium of cell cultures that can transfer information to recipient cells. Exosomes are involved in intercellular communication in physiology and disease. They are characterized by the size of 30–100 nm in diameter and an endocytic origin, formed by the reverse budding of the multivesicular bodies and released upon their fusion with the plasma membrane (Thery et al., 2002; Meng et al., 2013). More recently, exosomes derived from immunosuppressive dendritic cells (DC) have been found to confer potent and lasting immunosuppressive effects, like their parental DC. Recent progress suggests that exosomes hold a great promise in serving as potential novel therapeutics for inflammations (Buzas et al., 2014), cardiovascular diseases (Boulanger et al., 2017), metabolic diseases (Safdar et al., 2016; Shi et al., 2017) and cancers (Whiteside, 2017). However, an important question remains whether LIUS could make use of natural exosomes as a mechanism underlying its anti-inflammatory effects.

Therefore, we utilized an extensive data mining strategy on publicly available databases to better understand the potential molecular mechanisms that may be involved in LIUS mediated anti-inflammatory effects. Most interestingly, our analyzed data revealed several novel mechanisms that may potentially be attributed to protective effects exerted by LIUS. We found that LIUS can modulate anti-inflammatory gene expression, increase the immunosuppressive markers and master regulators which indicate increased number and activity of immunosuppressive cells, increased biogenesis and docking of exosomes that carry anti-inflammatory/immunosuppressive molecules. Our results have provided novel insights on how ultrasound inhibits inflammation and lay a novel mechanistic foundation for the development of novel ultrasound therapy for treatment of cancers, inflammatory diseases, tissue regeneration, and tissue repair.

## Methods

**Figure 1:**
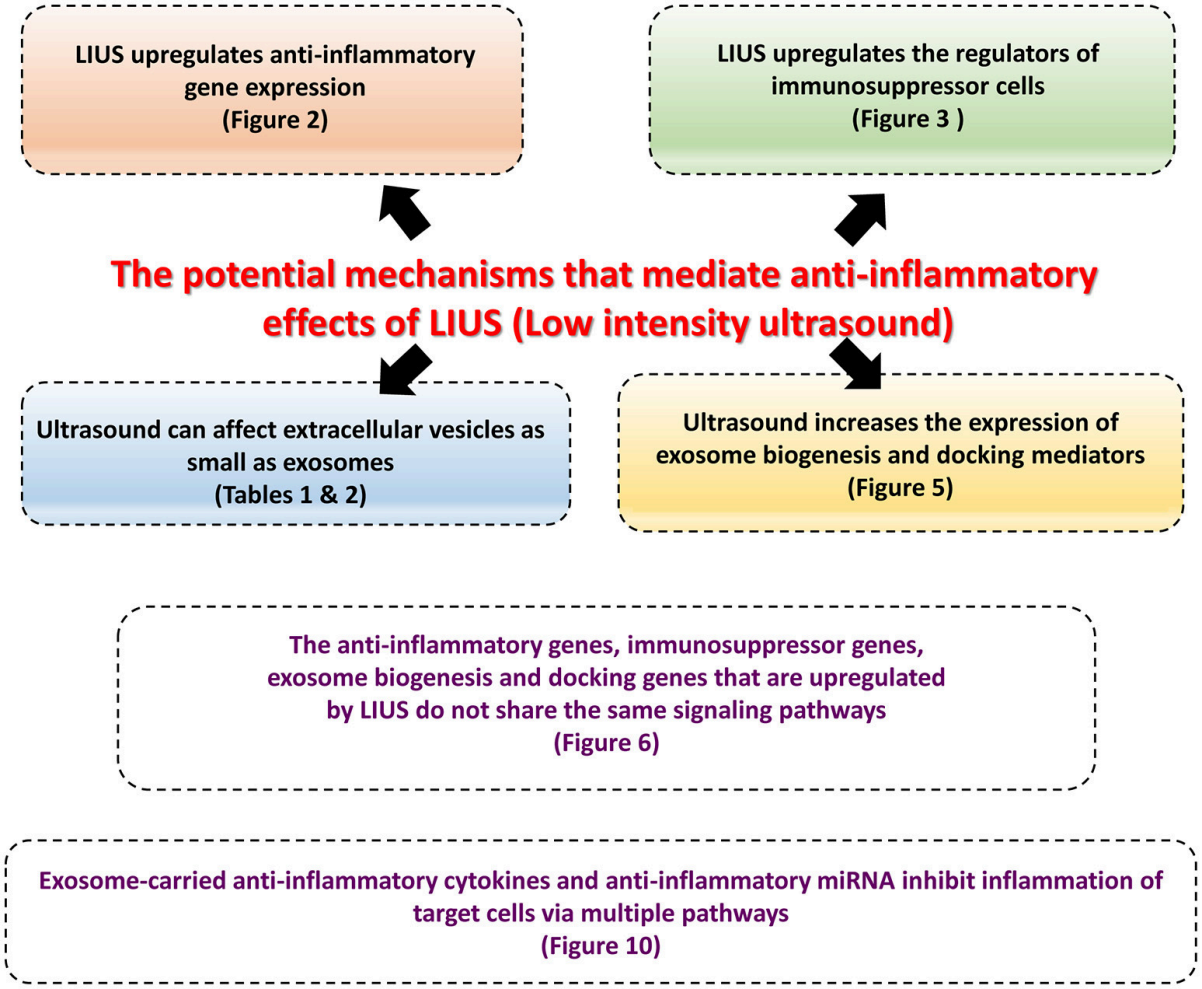
The logic flow and the overview of the manuscript.

We have utilized a well-established data mining strategy that we originally reported in 2004 (Ng et al., 2004; Yin et al., 2009; Li et al., 2012; Shao et al., 2016) to identify the potential molecular mechanisms that are responsible for exerting anti-inflammatory effects of ultrasound (Figure 1).

### Expression profile of ultrasound-induced genes

Gene expression profiles of microarray datasets conducted on ultrasound- treated tissues/cells were analyzed. These datasets were extracted from National Institutes of Health (NIH)-National Center for Biotechnology Information (NCBI) GEO Profile and NIH-NCBI-GEO DataSet databases (https://www.ncbi.nlm.nih.gov/geo/) and published papers with non-NIH-NCBI-Geo-deposited microarray data conducted on ultrasound-treated cells (Tabuchi et al., 2007; Hundt et al., 2008; Lu et al., 2009). Specific samples were chosen as ultrasound treatment groups and parallel controls. The number of samples was always greater than three except for the pooled samples.

First, we examined the expression of three house-keeping genes including Rho GDP dissociation inhibitor alpha (ARHGIDA), glyceraldehyde-3-phosphate dehydrogenase (GAPDH), and ribosomal protein S27a (RPS27A) as we reported previously (Yin et al., 2009). The microarray datasets (GDS3196) that we found in the NIH-NCBI-Geo DataSet database was conducted on non-ultrasound-treated cells vs. ultrasound-treated cells. This dataset had the mean ± 2 times standard deviations (SD) of the three house-keeping genes at X ± 2 SD = 1.00 ± 0.06 for the two groups, suggesting that the microarray experiments in Tabuchi et al. (2007) report were well performed; and that the dataset was trustable to be used for our analysis. Then, we selected the genes with statistically significant expression changes (*p* < 0.05) in the microarray data set and examined the fold change of the genes of our interest. Second, the genes with more than one-fold expression change were defined as the upregulated genes while genes with their expression change less than one-fold were defined as downregulated genes.

### Anti-inflammatory molecules in exosomes

We analyzed experimentally verified anti-inflammatory microRNAs (miRNAs) in the Exocota exosome database (http://www.exocarta.org).

### Molecular interaction network analysis

We used the Cytoscape software (http://www.cytoscape.org/) platform to visualize molecular interaction networks and biological pathways before we searched for detailed pathways with Ingenuity Pathway Analysis.

### Ingenuity pathway analysis

We utilized Ingenuity Pathway Analysis (IPA, Ingenuity Systems) (https://www.qiagenbioinformatics.com/) to characterize clinical relevance, and molecular and cellular functions related to the identified genes in our microarray analysis. The differentially expressed genes were identified and uploaded into IPA for analysis. The core and pathways analysis was used to identify molecular and cellular pathways as we have previously reported (Wang et al., 2016; Li et al., 2017).

### MicroRNA (miRNA) experimentally-identified target database

We analyzed the numbers of experimentally-identified mRNA targets for each microRNA (miR) in the microRNA database (http://mirtarbase.mbc.nctu.edu.tw/php/search.php) (Chou et al., 2016).

## Results

### Low-intensity ultrasound (LIUS) anti-inflammatory effects are mediated by upregulating anti-inflammatory gene expression

**Figure 2:**
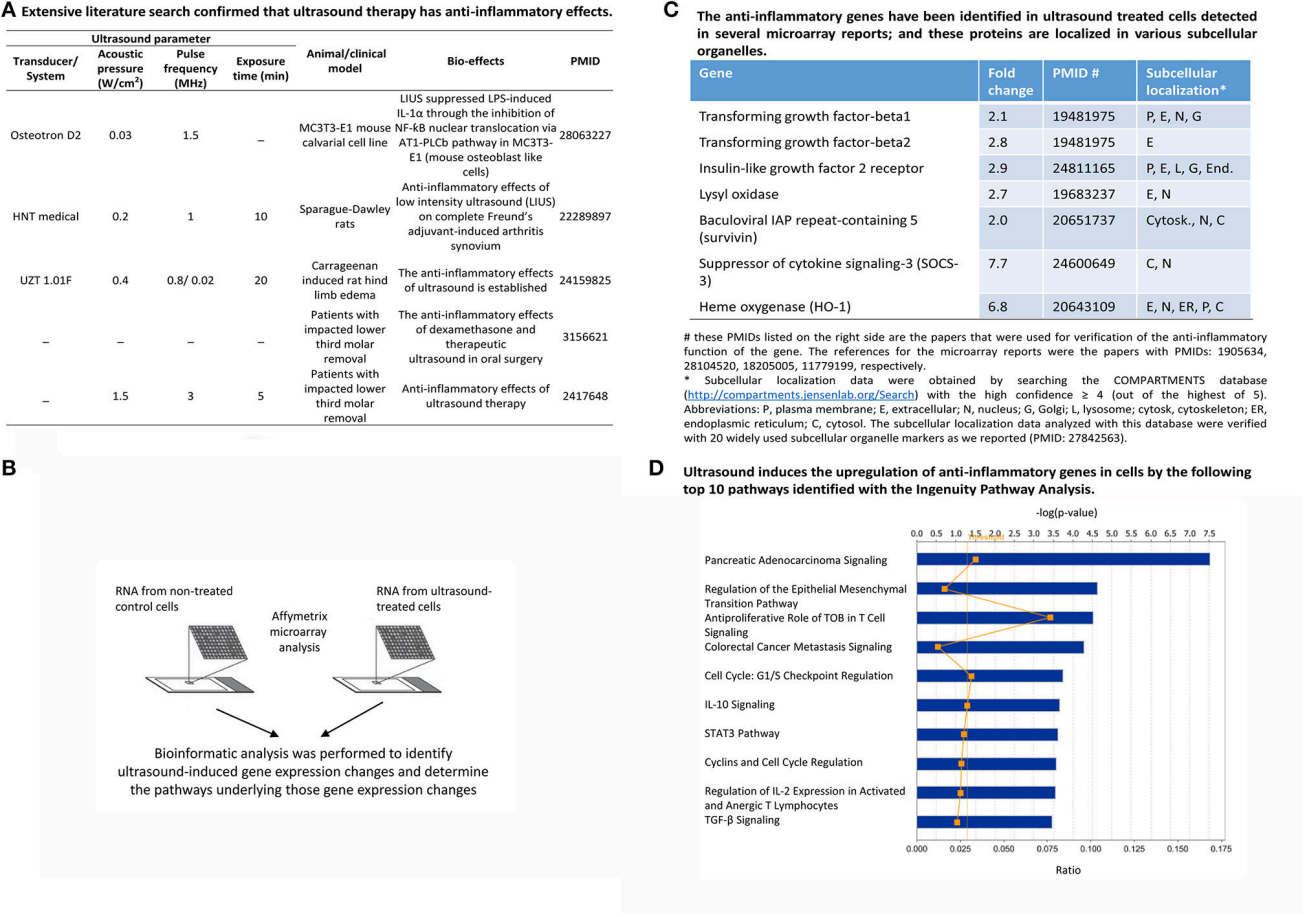
LIUS exerts anti-inflammatory effects in various cell-, animal- and clinical models by upregulating anti-inflammatory gene expression. **(A)** An extensive literature survey confirmed that ultrasound therapy exerts anti-inflammatory effects. **(B)** Schematic representation of the logic behind the microarray analysis we conducted to determine the anti-inflammatory gene expression. **(C)** The list of anti-inflammatory genes that are upregulated with LIUS, revealed by our data analysis. **(D)** The major signaling pathways regulated by the anti-inflammatory genes that are upregulated by LIUS.

Many publications have shown that LIUS exerts multiple biological functions including anti-inflammatory effects. The physical effects such as heat, shock wave and shear force that are created especially by microbubble cavitation in insonated fluid was attributed to these biological events. Through our extensive literature search shown in Figure 2A, we found that the first report of the anti-inflammatory properties of LIUS indicated its potential clinical use in reducing postoperative morbidity in oral surgery (ElHag et al., 1985). Therapeutic LIUS is used extensively in clinics to treat a wide variety of soft-tissue injuries. It is reputed to reduce swelling, pain and to accelerate tissue repair (Nagao et al., 2017). A recent report demonstrated that LIUS inhibits lipopolysaccharide (LPS)-induced interleukin-1α (IL-1α) via angiotensin II receptor type 1 (AT1)-phospholipase-Cβ (PLCß) pathway in osteoblasts (Nagao et al., 2017). In addition, use of LIUS treatment in inflammatory process facilitates the pathologically elevated whole protein levels to be brought back to physiological levels. Moreover, anti-inflammatory effects of LIUS are closely related to the decrease of inflammatory cell infiltration in the synovium and attenuation of hyperplasia (Chung et al., 2012). Furthermore, it is established that both therapeutic ultrasound and ultrasound given in lower intensity can exert anti-inflammatory effects but the two modalities differ in the subcellular mechanisms by influencing the cytosol and mitochondrial cell structures differently (Kravchenko et al., 2013). However, as mentioned above, the molecular mechanisms regarding ultrasound-induced anti-inflammatory effects remain poorly characterized.

Although previous microarray analysis results showed that LIUS modulates the gene expression in several cell types (Tabuchi et al., 2007; Hundt et al., 2008; Lu et al., 2009), the important issue on whether LIUS induces anti-inflammatory gene expression remains to be addressed. Therefore, we hypothesized that LIUS has anti-inflammatory effects in various tissues and cells, which are mediated by upregulating anti-inflammatory gene expression. To examine this hypothesis, we conducted an extensive literature search to find relevant microarray datasets (Figure 2B) and first compiled a list of seven ultrasound-induced anti-inflammatory genes with significant expression changes (>2.0-folds) (Figure 2C; Tabuchi et al., 2007; Hundt et al., 2008; Lu et al., 2009). In addition, our literature survey revealed that ultrasound also downregulated three major histocompatibility complex (MHC)-related highly significant immunogenic/pro-inflammatory genes with the decreased fold changes from −5.9 to −32.5 (Hundt et al., 2008) (not shown). This evidence substantiated our hypothesis that ultrasound exert its anti-inflammatory effects by downregulating pro-inflammatory genes and upregulating anti-inflammatory genes.

Then, we performed Ingenuity Pathway Analysis (IPA) to investigate the potential cellular mechanism pathways regulated by the seven anti-inflammatory genes that we identified and shown in Figure 2C. Ingenuity Pathway Analysis (IPA) database have integrated all the signaling pathway information extracted from the published literature and is a good resource that can be used to distinguish integrated cellular mechanisms regulated by a set of genes of interest (Thomas and Bonchev, 2010). Our IPA analysis revealed that LIUS mediated activation of anti-inflammatory genes can activate multiple anti-inflammatory pathways, including anti-proliferative role of transducer of erbB-2/B-cell translocation gene (TOB) in T cell signaling, anti-inflammatory cytokine interleukin-10 (IL-10) signaling, immunosuppressive/anti-inflammatory CD4+ regulatory T cell (Treg) surviving factor IL-2, and anti-inflammatory cytokine transforming growth factor-β (TGF-β) signaling (Figure 2D).

Furthermore, since protein function is related to its subcellular localization (Yu et al., 2006), we hypothesized that LIUS induces the global effects of anti-inflammatory proteins by inducing protein localized in various subcellular organelles. As we reported previously, by confirming all the subcellular localizations of 21 common used subcellular organelle markers, we concluded that widely-used subcellular localization database COMPARTMENTS is reliable (Wang et al., 2016). The results showed in the right most panel of Figure 2C indicate that the subcellular localization of seven ultrasound-induced anti-inflammatory molecules TGF-β1 and TGF-β2, the prototypic anti-inflammatory cytokines, are found in the extracellular (E) (Li et al., 2012; Shao et al., 2014), insulin-like growth factor 2 receptor and heme oxygnerase are found in the plasma membrane and suppressor of cytokine signaling-3 (SOCS-3), surviving and lysyl oxidase are found in both cytosol and nucleus. The majority of seven proteins had multiple subcellular localization. The results showed that LIUS has global cellular functions by upregulating proteins with various subcellular localizations to fulfill their pleiotropic effects of inflammation inhibition, similar to that we reported for a different pathological process (Fu et al., 2017). Our results clearly shows that LIUS inhibits inflammation by activating anti-inflammatory genes; and others have previously reported that LIUS can suppress certain pro-inflammatory genes substantially (Hundt et al., 2008). Therefore, altogether it is shown that LIUS inhibits inflammation by both inhibiting immunogenic/pro-inflammatory genes and promoting anti-inflammatory gene expression in order to play important roles in anti-inflammatory cellular functions.

### Ultrasound induces the upregulation of the regulators of immunosuppressor cells including myeloid-derived suppressor cells (MDSCs), mesenchymal stromal/stem cells (MSCs), B1-B cells and CD4+Foxp3+ regulatory T cells (Treg)

**Figure 3:**
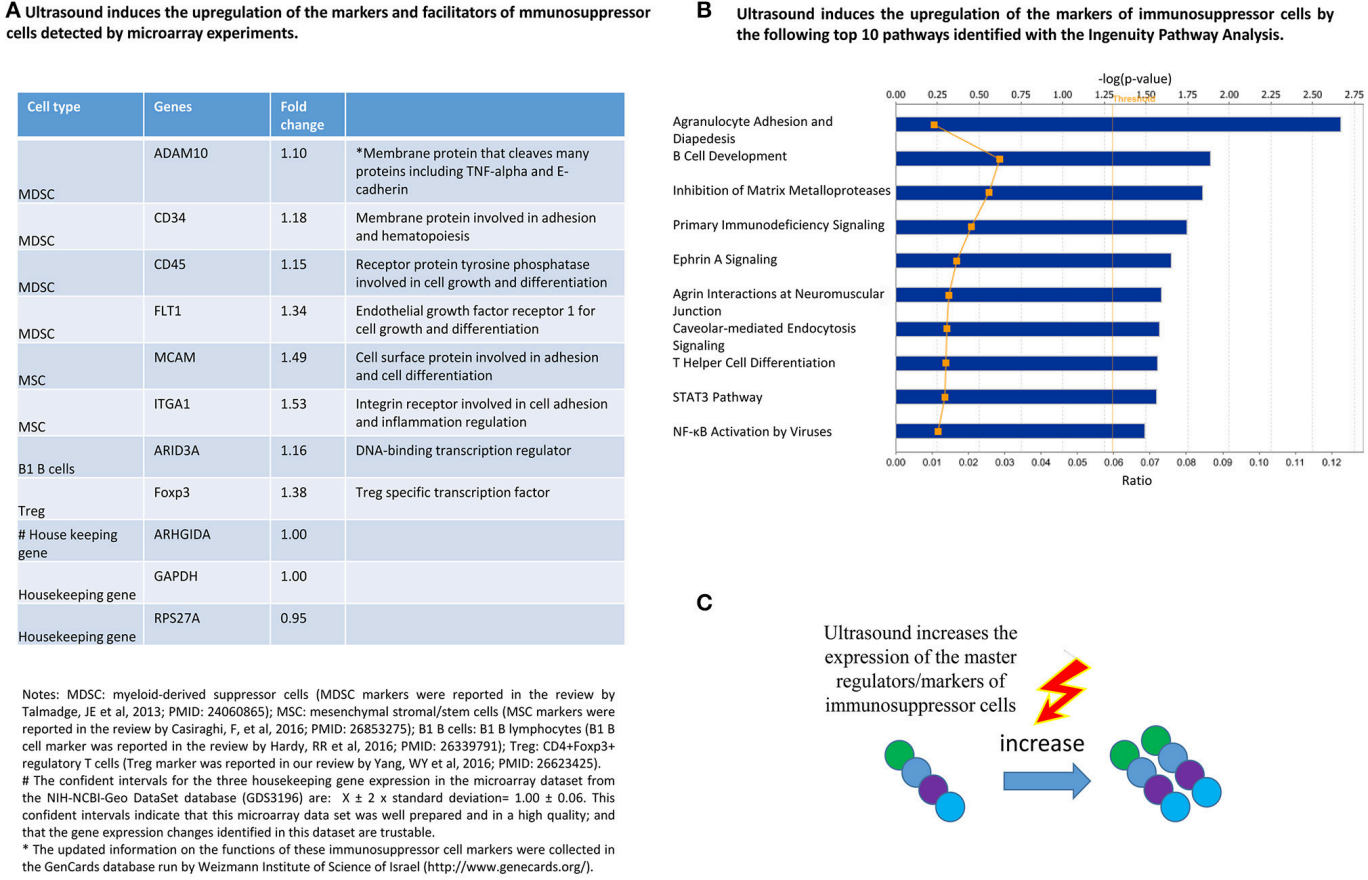
LIUS therapy increases the expression of immunosuppressive cell markers/regulators. **(A)** The list of regulators of immunosuppressive cells that showed increased expression when subjected to LIUS. **(B)** The major signaling pathways that are affected by the regulators of immunosuppressive cells identified in our analysis. **(C)** Graphical representation of our conclusion that LIUS may increase the expression of markers/regulators of immunosuppressive cells.

We then hypothesized that in addition to LIUS's anti-inflammatory molecular and cytokine mechanisms, ultrasound may further strengthen its anti-inflammatory effects by inducing the generation/development of immunosuppressor cells as cellular mechanisms. To test this hypothesis, we tried to find the supporting evidence of gene upregulation using the microarray data analysis collected from LIUS-treated cells in the NIH-NCBI-Geo DataSets (GDS3196). First we validated the datasets as described in the methods section and then examined the expression changes of three MDSC markers such as ADAM metallopeptidase domain 10 (ADAM10), CD34, protein tyrosine phosphatase receptor type C (PTPRC, CD45) and vascular endothelial growth factor receptor 1 (VEGFR1, FLT1) (Talmadge and Gabrilovich, 2013), two MSC markers such as melanoma cell adhesion molecule (MCAM), and integrin subunit alpha 1 (ITGA1) (Casiraghi et al., 2016), one CD5+ B1 B cell (Tsiantoulas et al., 2015) transcription factor and marker AT-rich interaction domain 3A (ARID3A) (Hardy and Hayakawa, 2015), and one Treg transcription factor forkhead box P3 (Foxp3) as we reported (Yang et al., 2015) in the dataset of LIUS-treated cells. The results in Figure 3A shows that LIUS increased the expression of those genes significantly up to 1.53-folds. Although the upregulation of those immunosuppressor cell regulator genes induced by ultrasound were in low folds, the fold increases of those genes were significantly higher than the house-keeping gene confident intervals (*p* < 0.05) (Figure 3A), suggesting that these LIUS-induced upregulation are statistically significant (Dalman et al., 2012). Although this dataset was not generated from specific immunosuppressor cells, it was important to document the significant effects of ultrasound in specifically inducing the immunosuppressor cell markers. Of note, these eight immunosuppressor cell molecules are not the only markers for being used in the experiments to identify these cells, these markers are master genes with multiple regulatory functions in defining the immunosuppressive roles of these cells. For example, ARID3A is a key transcription factor for B1 B cells (Hardy and Hayakawa, 2015); and Foxp3 is a transcription factor in determining the biogenesis and immunosuppressive function of Tregs (Pastrana et al., 2012; Yang et al., 2015). Deficiency of Foxp3 leads to failed development of Tregs (Yang et al., 2015); and the levels of Foxp3 in Tregs reflect their functional status (Chauhan et al., 2009), suggesting that LIUS-induced Foxp3 expression enhances Treg immunosuppressive function. Therefore, a slight increase in the expression of these master regulators are sufficient to exert significant impact on cellular and biological functions.

We also performed IPA (Figure 3B) and found that LIUS induces the upregulation of immunosuppressor cell regulators that can profoundly affect well-characterized anti-inflammatory pathways including inhibition of matrix metalloproteases, primary immunodeficiency signaling, caveolae-mediating endocytosis signaling, and T helper cell differentiation including Treg. Once again, these results showed that the LIUS-induced upregulation of immunosuppressor cell master regulators promote the development and functions of immunosuppressor cells (Figure 3C).

### LIUS induces the expression of extracellular vesicle/exosome biogenesis mediators and extracellular vesicle/exosome docking mediators

There are multiple reports substantiating that ultrasound microbubble-mediated cavitation enhance nanoparticle and liposome-mediated drug delivery to target tissues (Table 1). Furthermore, some reports attribute the sonoporation effect to enhanced drug delivery to target tissues (Table 2). All these reports claim that sonoporation effect and ultrasound-mediated microbubble cavitation can facilitate perforation, membrane blebbing and drug/gene delivery in various sizes of membrane structures similar to exosomes. For example, Lin et al. used four different sizes of lipid-coated CdSe quantum dot (LQD) nanoparticles ranging from 30 to 180 nm, 1.0-MHz pulsed focused ultrasound (FUS) with a peak acoustic pressure of 1.2-MPa, and an ultrasound contrast agent (UCA; SonoVue) at a dose of 30 μl/kg; and found that FUS-induced UCA oscillation/destruction results in rupture areas in blood vessels increasing the vascular permeability and enhances targeted delivery in tumors (Lin et al., 2010). These reports demonstrated that ultrasound has physical capacity in affecting membrane vesicles as small as exosomes (30–100 nm diameters) suggesting that ultrasound may have an impact on exosomes and unload its cargo in to the insonated media facilitating cellular uptake. Therefore, it can be suggested that ultrasound not only can be used as novel therapeutic approaches to deliver drugs packed in various structures such as nanobeads, nanospheres, polymer microspheres and lipidosomes, but also could make use of natural exosomes derived from immunosuppressor cells as a mechanism to fulfill its anti-inflammatory effects.

**Table 1:** Numerous publications have reported that the microbubble-mediated ultrasound cavitation enhances nanoparticle delivery in cultured cells and experimental animal models.

**Ultrasound parameter**					**Micro-bubble**	**Nanoparticle**	**Size (nm)**	**Cell**	**Animal model**	**Bioeffects**	**PMID**
**Trausducer/System**	**Acoustic pressure**	**Pulse fre-quency (MHz)**	**Pulse repetition frequency**	**Expo-sure time (s)**							
_	1.82 Mpa	1	100 MHz	60	Avidin-AuMBs		2.8	Colon cancer cells	BALB/c mice	Ultrasound-induced MB disruption assists the cellular delivery of AuNRs	25023090
A392S	1.2 Mpa	1	1 Hz	120	SonoVue	Lipid coated quantum dots	30, 80, 130, 180	CT-26 cells	BALB/c mice	Ultrasound-induced MB significantly enhance the delivery of LQD nanoparticles into tumor tissues	20621645
Spherically	1.9 Mpa	2.25	0.25 Hz	120	Lipid-biotinylated	Lipidsomes	100, 200	PC-3 cell monolayer	_	Alternate methods of microbubble–liposome conjugation, the therapeutic response and the in vivo performance of the liposome-bearing microbubbles are now under evaluation	17300849
HDI 3000cv	_	2.3	_	_	Optison	Polymer microspheres	205, 503	–	Sprague-dawley rats	Delivery of Colloidal Particles and Red Blood Cells to Tissue through micro-vessel Ruptures	9751673
Cylindrically focused single element	0.75 Mpa	1	_	_	Optison	Polymer microspheres	100	–	Sprague-dawley rats	The ultrasound PI and microvascular pressure significantly influence the creation of extravasation points and the transport of microspheres to tissue	11849875
Sonos 5500	1.8 Mpa	1.3	_	_	PSEDA	Fliorescent nanospheres	30, 100	–	Wistar rats	UTMD allows colloid nanoparticles to be delivered to the rat myocardium through micro-vessel rupture sites	16166101
Panametrics V305	1.1 Mpa	3	_	1.3	Lipid-biotinylated	Nanobead (neutravidin coated latrx beads)	40, 200	–	Cellulose tube	Enable targeted deposition of nanoparticles in shear flow and could be modified to carry therapeutic agents for controlled release in targeted delivery applications	16380187
Sonitron 2000	2 W/cm^2^	1	_	10	Lipid-biotinylated	Lipidsomes-siplex	120	HUH7 and HUH7eGFPLuc cells	–	Ultrasound assisted siRNA delivery using PEG-siPlex loaded microbubbles	18237813
_	_	1	_	150	Albumin	Polystyrene or PLGA	100	_	C57BL/6J mouse hind limb	Targeted Delivery of Nanoparticles Bearing Fibroblast Growth Factor-2 by Ultrasonic Microbubble Destruction for Therapeutic arteriogenesis	18720443
Sonitron 2000	2 W/cm^2^	1	–	10	Lipid-biotinylated	Cationic liposomes, lipolexes, siPLEXES	125-325	BLM, HuH-7, HUH7eGFPLuc, A549, Vero cells	_	New strategies for nucleic acid delivery to conquer cellular and nuclear membranes	18655814
Sonitron 2000	2 W/cm^2^	1	_	15	Lipid-biotinylated	Lipidsomes, doxorubicin	147	BLM cells	–	DOX- liposome- loaded microbubbles could be a very interesting tool to obtain an efficient ultrasound-controlled DOX delivery in vivo	19623162
Unfocused 0.75 in diameter	075 Mpa	1	–	5	Albumin	PLGA	150		BALB/c mouse hind limb	Covalently linking 150 nm-diameter poly(lactic-co-glycolic acid) nanoparticles to microbubbles before intravenous injection does improve their delivery to skeletal muscle	21456081

**Table 2:** Many publications have reported that the sonoporation and effects of microbubble-mediated ultrasound cavitation facilitate perforation, membrane blebbing and drug/gene delivery in various sizes of membrane structures including exosomes.

**Ultrasound parameter**				**Microbubbles**			**Model**	**Bioeffects**	**PMID**
**Transducer/System**	**Acoustic pressure (Mpa)**	**Pulse frequency (MHz)**	**Exposure time**	**Name**	**Concentration (bubbles/ml)**	**Size (μm)**			
Sonopore 4000	0.12	0.834	50 ms	Sonazoid	0.6 × 10^9^ ± 5%	2.6	Human lymphoma cells (U937)	Overstretched cell membrane causes reparable submicron pore formation	26941839
Single-element unfocused piston transducer	0.45	1		Perfluoropropane (C3F8) gas	1 × 10^8^	1–5	Human breast carcinoma cells (CCL-1504)	Membrane blebbing would help sonoporated cells restore homeostasis	25694544
Singleelement piston transducer	0.45	1	60 min	Lipid-shelled	1 × 10^8^	2–4	Human breast carcinoma cells (CCL-1504)	Sonoporation as an emerging membrane perforation technique/organization of the actin cytoskeleton is concomitantly perturbed	24671936
A dual-frequency transducer assembly	1.4	7.44	40 μs	Laser induced microbubbles	_	3–15	Xenopus oocytes	A combined approach synchronized manipulation, imaging, and measurement of cavitation of single bubbles and the resulting cell membrane disruption in real-time.	21945682
_	0.05–3.5	0.5–5.0	0.1 - 900 s	Definity	1.2 × 10^6^	1–8	Murine fibrosarcoma cell line KHT-C cells	Acoustic exposure parameters on cell membrane permeability and cell	19110370
_	0.05–3.5	1.075	0.2 s	Definity	6 × 10^3^	1–8	Xenopus laevis oocytes	Pore size obtained from the TMC measured using the voltage clamp technique	19647924
Sonos 5500	_	3.6	10 s	SonoVue	2 × 10^8^	2.5	H9c2 rat cardiomyoblast cells	Transient permeabilization of cell membranes by ultrasound-exposed microbubbles	16632548
Sofranel	150	1	_	SonoVue	2 × 10^8^	1–12	Mammary breast cancer cell line MDA-MB-231	Cell electrophysiological properties is a necessary toward understanding mechanisms of cell membrane permeabilization	17189059
Co-linear array	0.2	1.4	2 min	Lipid-biotinylatedr	10^8^–10^10^	1–10	Mouse thigh muscle model	Local microbubble-enhanced sonoporation of plasmid DNA. With the aim of optimizing delivery efficiency	26682505
Nexus	100 mW/cm^2^	0.0465	12 h	Sonovue	2–5 × 10^8^	2.5	Escherichia coli (ATCC 25922)	Enhance the bactericidal effect and cause partial destruction of the bacterial cell wall	24977141
RFG1000	2.7	1.1	90 s	Lipid-shelled	2–5 × 10^9^	0.6–18	Human embryonic kidney 293T cells/Rats	Enhancing Gene Delivery	24650644
Single element planar transducer	0.4–1.6 a	1.25	8 μs–10 ms	Targestar™-SA	1 × 10^9^		Human umbilical vein endothelial cells (HUVECs)	Improve sonoporation gene transfection and delivery	23770009
Imasonic	0.88	1.5	30s	Sonovue	2 × 10^8^	2.5	ATCC C6 rat glioma cells	Ultrasound-Mediated drug delivery	22707046
Single planar circular transducer	0.24	1.25	_	Definity	1 × 10^6^	1.1–3.3	bEnd.3 cells/immortalized mouse cell line	Generate immediate [Ca2+]i changes in brain microvascular endothelial cells	20620704
_	0.05–3.5	0.5–5.0	0.1–900 s	Definity	1.2 × 10^6^	1–8	Murine fibrosarcoma cell line KHT-C cells	Acoustic exposure parameters on cell membrane permeability and cell	19110370
V303	0.1–0.5	1	30 s	Sonovue	2 × 10^8^	2.5	H9c2 rat cardiomyoblast cells	Local hyperpolarization of the cell membrane via activation of BKCa channels	17993242
Panametrics	0.4	1	5 s	BR14	5 × 10^8^	1–2	Bovine endothelial cell (ATCC)	Drug transfer into cells via sonoporation	16556469
Air-backed transducer	0.402–0.507	1.15	10 s−2 min	Bracco	25–30 particles/cell		Rat mammary carcinoma cells (MAT B III)	Direct transfer of 37 nm macromolecules into the cytoplasm	15866347
A circular planar	0.6	0.96–1.2	0.5 s	Albumin-shelled (C3F8) gas		3.2	Xenopus oocyte	Ca2^+^ entering the cell through US-induced pores	15121254
Panametrics	0.9	1	30 times	Sonovue	2 × 10^8^	1–12	Pig aortic endothelial cell	Camera makes it possible to reveal the mechanisms of interactions between ultrasound, microbubbles and cells	15550330
_	2.5 W/cm^2^	1	30 s	Optison	_	_	Human skeletal muscle cells	Ultrasound: enhancement of transfection efficiency of naked plasmid DNA in skeletal muscle	11960313

**Figure 4:**
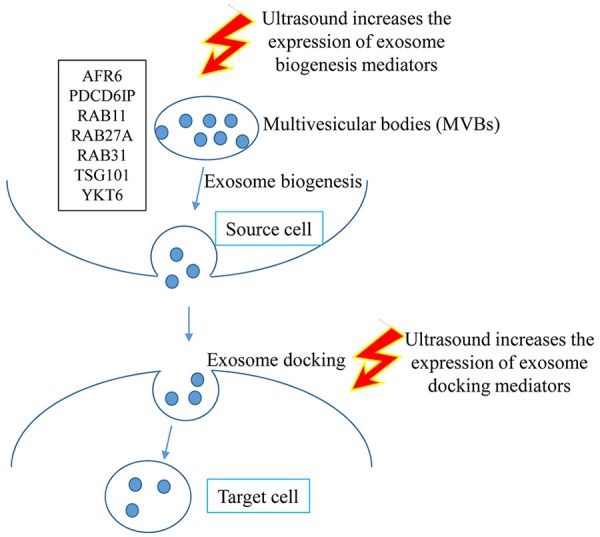
Graphical representation of the hypothesis that LIUS may increase exosome biogenesis and docking in the tissues exposed to ultrasound therapy.

After taking all these facts in to consideration, we formulated a new hypothesis that ultrasound may affect the biogenesis of exosomes and its functions (Figure 4). Exosomes are a type of membrane vesicles secreted by various cell types. Exosomes are characterized by a size of 30–100 nm in diameter, and are formed by the reverse budding of multivesicular bodies and released via their fusion with the plasma membrane (Yang and Robbins, 2012).

**Figure 5:**
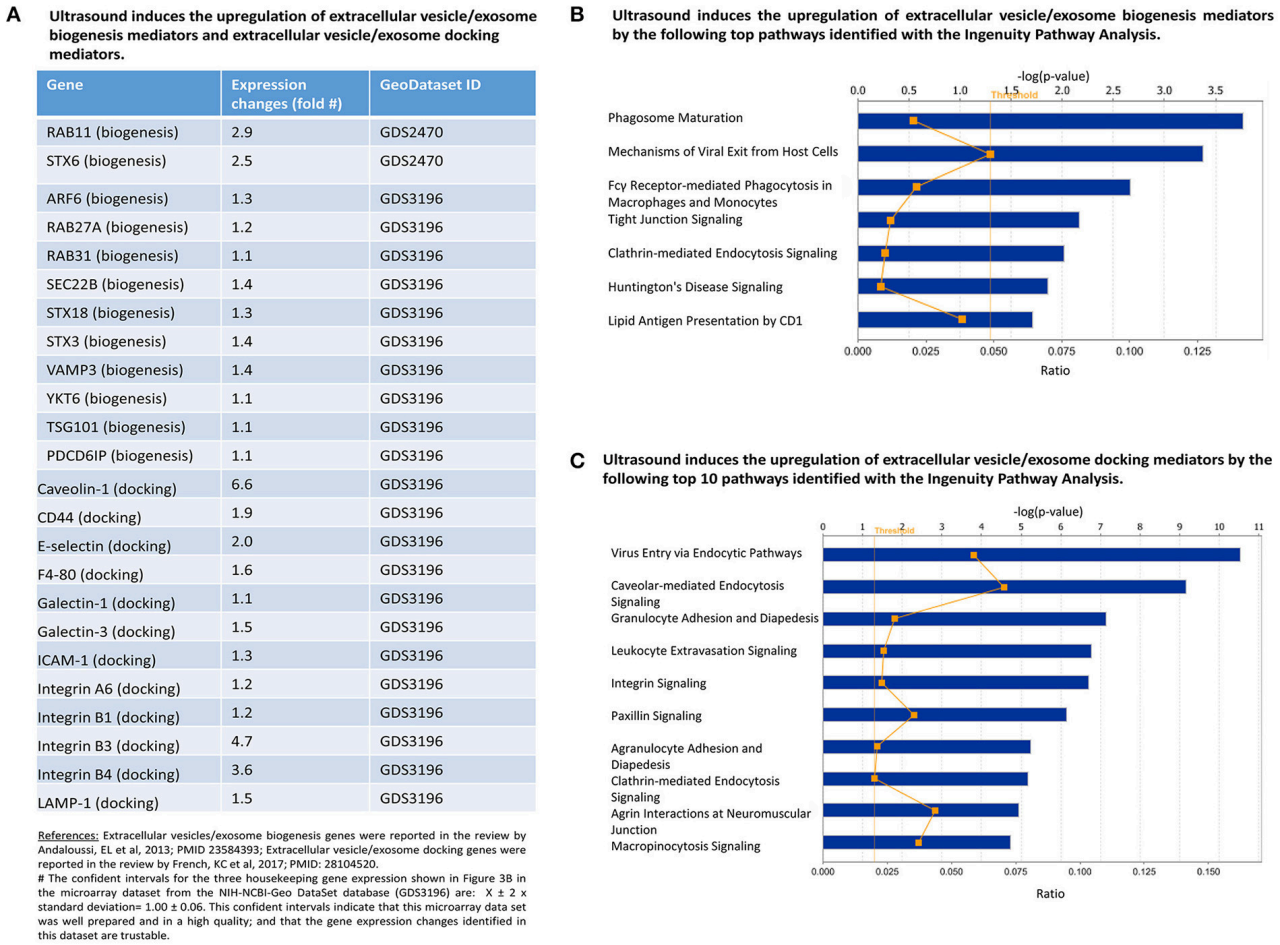
LIUS therapy increases markers of exosome biogenesis and docking**. (A)** List of exosome biogenesis and docking genes that were upregulated with LIUS therapy. **(B)** The signaling pathways that are affected by the exosome biogenesis genes that are upregulated with LIUS treatment. **(C)** The major signaling pathways that are regulated by the exosome docking genes that had increased expression with LIUS therapy.

To test our hypothesis, we examined the expression of 12 reported extracellular vesicle/exosome biogenesis mediators (Meng et al., 2013) and also 12 reported extracellular vesicle/exosome docking mediators (French et al., 2017) in LIUS-treated cells in the microarray dataset. As shown in Figure 5A, LIUS significantly induced the expression of 12 extracellular vesicle/exosome biogenesis mediators up to 2.9-folds (RAB11, a small GTPase signal transducer for vesicle trafficking; Campa and Hirsch, 2017) and 12 docking mediators up to 6.6-folds (caveolin-1, a plasma membrane invagination mediator for clathrin-coated pits and calveolae; Shankar et al., 2015). We also performed the IPA on the exosome biogenesis genes that have increased expression levels when exposed to LIUS (Figure 5B). Of note, since exosomes as intercellular communication events are secreted by most cell types (Thery, 2011), our finding of LIUS upregulation of exosome biogenesis and docking regulators is not limited to the cell type used in the original microarray experiments and can also be applied to immunosuppressor cells. Our analysis revealed that these genes can significantly impact and regulate; phagosome maturation, mechanisms of viral exit from host cells, Fcγ receptor-mediated phagocytosis in macrophages and monocytes, tight junction signaling, clathrin-mediated endocytosis signaling, Huntington's disease signaling, and lipid antigen presentation by CD1. Similarly, we conducted IPA on exosome docking genes that are upregulated by ultrasound exposure (Figure 5C). We found that these genes can regulate endocytic, caveolar-mediated endocytosis, granulocyte adhesion and diapedesis, leukocyte extravasation, integrin, paxillin, agranulocyte adhesion and diapedesis, clathrin-mediated endocytosis, agrin interactions at neuromuscular junction, and macropinocytosis.

There are ample evidence to show that various immune cell-, immunosuppressor cell-derived exosomes have anti-inflammatory effects (Table 3). The anti-inflammatory effects of exosomes have been demonstrated in arthritis, autoimmune disease, sepsis, colitis, neurodegenerative disease, diabetic wound healing, neuroinflammation, injury-induced inflammation, brain infarct zone, traumatic brain injury, myocardial infarction, induction of anti-inflammatory cytokines and Treg, inhibition of pro-inflammatory cytokines, and hepatitis C viral infection, etc. These results suggest that the LIUS-induced upregulation of extracellular vesicle/exosome biogenesis mediators and extracellular vesicle/exosome docking mediators may promote the anti-inflammatory effects of immunosuppressor cells-derived exosomes.

**Table 3:** A long list of publications have reported that various immune cell-, and immunosuppressor cell-derived exosomes have anti-inflammatory effects.

**Source of exosomes**	**Cell origin**	**Function**	**PMID**
DCs	Spleen DCs	SDC-expanded Tregs could inhibit the production of inflammatory cytokines	27640806
DCs	Murine bone marrow	Overexpressing IDO are anti-inflammatory in collagen-induced arthritis	19180475
DCs	Mouse bone marrow	Suppression of inflammatory and autoimmune responses	15879146
DCs	Mouse bone marrow	Treatment of inflammatory and autoimmune diseases	16275099
DCs	Rat bone marrow	Attenuate the acute systemic inflammatory response in sepsis	19812118
DCs	Rat bone marrow	Down-regulate the inflammatory response in TNBS-induced colitis	20469967
MDSCs	Lewis lung adenocarcinoma cell line	Decreased inflammatory cell infiltration damage	26885611
Macrophage	Mouse macrophage cell line	Treatment of inflammatory and neurodegenerative disorders.	25836593
MSCs	Human umbilical cord	Alleviated inflammation and enhanced diabetic cutaneous wound healing.	26386558
MSCs	Human bone marrow	Reduced neuro-inflammation	27539657
MSCs	Human umbilical cords	Suppress injury-induced inflammation	27686625
MSCs	Mini-pigs abdominal adipose tissue	Reduce brain-infarct zone	27793019
MSCs	Human bone marrow	Significantly reduces brain inflammation in rats after TBI	27539657
MSCs	Mouse bone marrow	Improved the microenvironment of myocardial infarction through angiogenesis and anti-inflammation	26646808
MSCs	Rats bone marrow	Protect against experimental colitis via attenuating colon inflammation, oxidative stress and apoptosis	26469068
MSCs	huES9.E1 human embryonic stem cell	Attenuate an activated immune system through the induction of anti-inflammatory cytokines and Tregs	24367916
MSCs	Healthy donors' bone marrow	Suppresses the levels of the pro-inflammatory cytokine, IL-1β and TNF-α, but increases the expression of anti-inflammatory cytokine (TGF-β)	27115513
MSCs	Rats bone marrow	Improves functional recovery and promotes neurovascular remodeling (angiogenesis and neurogenesis) and reduces neuro-inflammation in rats after TBI	25594326
MSCs	Human umbilical cords	Exosomal MicroRNAs Derived From Umbilical Mesenchymal Stem Cells Inhibit Hepatitis C Virus Infection	27496568

### The anti-inflammatory genes, immunosuppressor regulator genes, exosome biogenesis and docking genes that are upregulated by ultrasound exposure do not share the same signaling pathways

**Figure 6:**
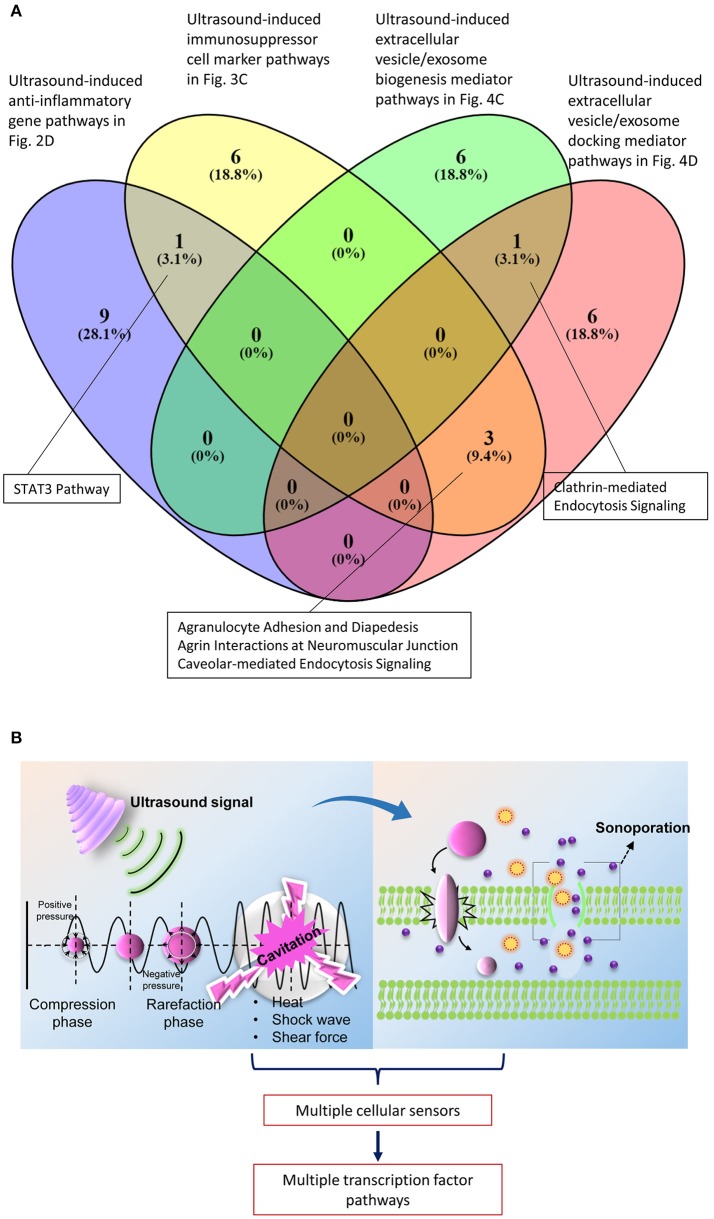
Most of the signaling pathways that are regulated by the mechanisms that are activated by LIUS are not shared. **(A)** Venn analysis revealed that STAT3 signaling pathway is shared between the anti-inflammatory genes and immunosuppressive regulator genes that are upregulated by LIUS therapy. Likewise, clathrin mediated endocytosis signaling is shared by genes that enhance exosome biogenesis and docking with ultrasound therapy and 3 signaling pathways are shared by immunosuppressor regulator genes and exosome docking genes. **(B)** The physical properties exerted by ultrasound therapy may activate multiple cellular sensors that activate distinct transcription factors to regulate various signaling pathways.

We further tested whether ultrasound mediated genes that regulate four main stream mechanisms we identified (anti-inflammatory gene upregulation—Figure 2, immunosuppressor regulator genes—Figure 3, exosome biogenesis and docking—Figures 5B,C) share same signaling pathways or not. To test this hypothesis, we used the Venn analysis as we reported previously (Li et al., 2017). Venn diagram (logic diagram) shows all possible logical relations between a finite collections of different data sets. The results in Figure 6A shows that each of the four LIUS-induced anti-inflammatory mechanisms we identified have their own specific pathways. We also found the following interesting results.

*First*, LIUS-induced anti-inflammatory gene upregulation (Figure 2D) shared STAT3 (signal transducer or activator of transcription 3) pathway with LIUS-induced immunosuppressor cell marker upregulation (Figure 3B); *second*, LIUS-induced immunosuppressor cell marker upregulation (Figure 3B) shared three pathways such as agranulocyte adhesion and diapedesis, agrin interaction at neuromuscular junction and caveolar-mediated endocytosis signaling with LIUS-induced extracellular vesicle/exosome docking mediator upregulation pathway (Figure 5C); and *third*, LIUS-induced extracellular vesicle biogenesis mediator upregulation (Figure 5B) shared one pathway of clathrin-mediated endocytosis signaling with LIUS-induced extracellular vesicle/exosome docking mediator upregulation pathway (Figure 5C). Once again, our results suggest that LIUS anti-inflammatory effects materialize via integrated and orchestrated efforts in inducing anti-inflammatory gene upregulation, immunosuppressor cell marker upregulation, extracellular vesicle/exosome biogenesis mediator upregulation and extracellular vesicle/exosome docking mediator upregulation. Also our data indicate that the most of the pathways regulated by these mechanisms are not shared with each other, suggesting that LIUS generated physical effects such as heat, shock wave and shear force (Izadifar et al., 2017) are sensed by multiple cellular sensors that activate various transcription factor pathways (Figure 6B); and that future optimization of LIUS anti-inflammatory effects need to use our newly identified molecular pathways as experimental readouts and test each of three physical effects including heat, shock wave and shear force.

### Exosome-carried anti-inflammatory cytokines and anti-inflammatory micoRnas inhibit inflammation of target cells via multiple shared and specific pathways

**Figure 7:**
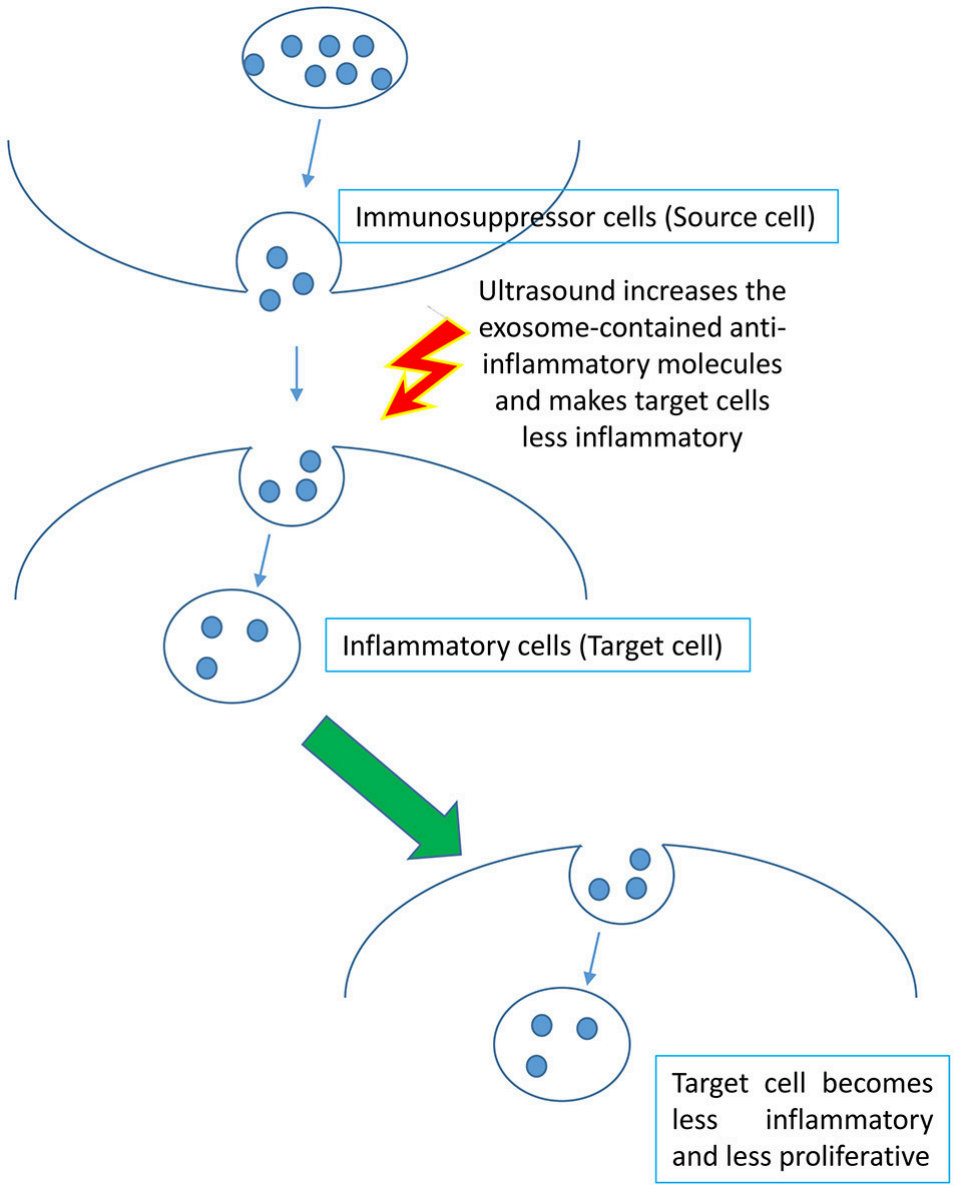
Schematic representation of the hypothesis that increased exosome biogenesis and docking release anti-inflammatory components to the insonated media and target cells resulting in suppression of inflammation.

**Figure 8:**
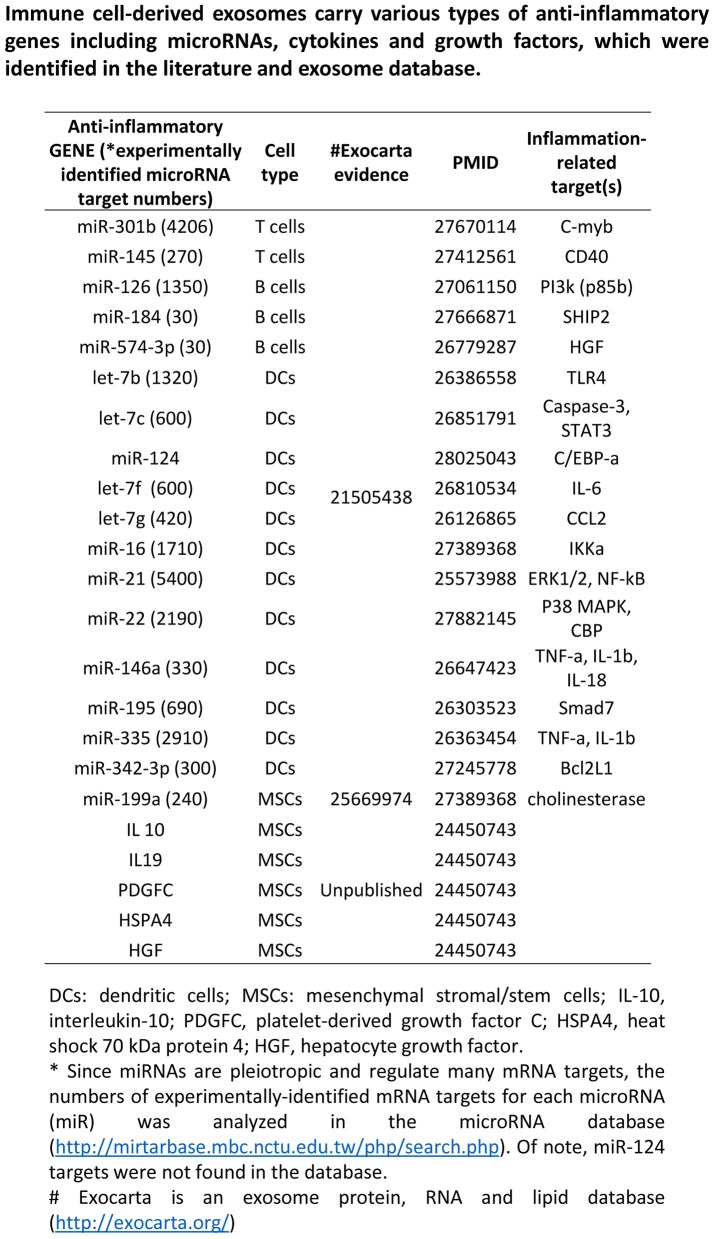
Immune cell derived exosomes carry anti-inflammatory miRNAs and cytokines. List of anti-inflammatory microRNAs, cytokines and growth factors carried by immune-cell derived exosomes.

**Figure 9:**
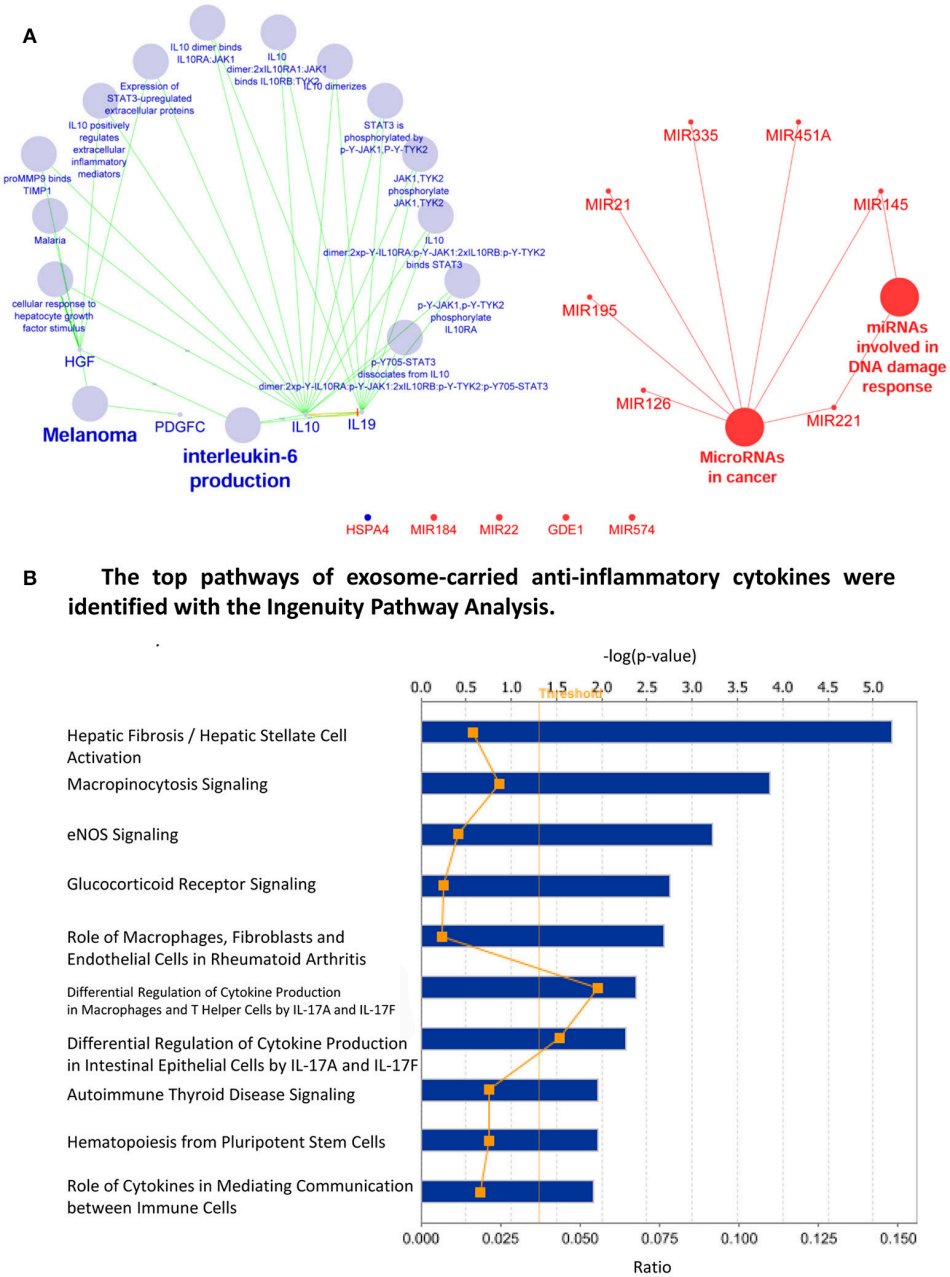
anti-inflammatory cytokines and miRNA found in immune cell derived exosomes have distinct cellular networks. **(A)** Cytoscape Network Visualization Analysis revealed that miRNA (red) and anti-inflammatory cytokine (green) have distinct cellular networks that are not shared (large spheres represent biological processes while small sphere indicates a gene/mRNA). **(B)** The major pathways that are regulated by the anti-inflammatory cytokines carried in exosomes derived from immune cells.

We then hypothesized that exosomes carry anti-inflammatory molecules and inhibit the inflammation of target cells through specialized pathways (Figure 7), which materialize the exosomes as an anti-inflammatory mechanism of LIUS. To test this hypothesis, we performed an extensive search for the evidence in the literature and the exosome database. As shown in Figure 8, we found 18 anti-inflammatory microRNAs (miRs) and 5 anti-inflammatory molecules/growth factors such as prototypic anti-inflammatory cytokine IL-10, IL-19, platelet-derived growth factor C (PDGFC), heat shock 70 protein 4 (HSPA4), and hepatocyte growth factor (HGF), in the exosomes derived from immune cells and immunosuppressor cells (Striz et al., 2014). To understand how the exosome-carried anti-inflammatory molecules inhibit the inflammation of target cells, we first determined whether anti-inflammatory miRs are connected in pathways with anti-inflammatory cytokines/growth factors. As shown in Figure 9A, we performed Cytoscape Network Visualization analysis to visualize the pathway connection between miRs and anti-inflammatory molecules we identified. Our data revealed that anti-inflammatory cytokines/growth factors and anti-inflammatory miRs are in different cellular networks. To further analyze the top 10 signaling pathways underlying the five anti-inflammatory cytokines/growth factors in exosomes shown in Figure 9B, we performed Ingenuity Pathway analysis. In Figure 9B, we showed that the five anti-inflammatory cytokines and growth factors use the following top 10 pathways: hepatic fibrosis/hepatic stellate cell activation, micropinocytosis signaling, eNOS signaling, glucocorticoid receptor signaling, role of macrophages, fibroblasts and endothelial cells in rheumatoid arthritis, differential regulation of cytokine production in macrophages and T helper cells, differential regulation of cytokine production in intestinal epithelial cells, autoimmune thyroid disease signaling, hematopoiesis from pluripotent stem cells and role of cytokines in mediating communication between immune cells.

**Figure 10:**
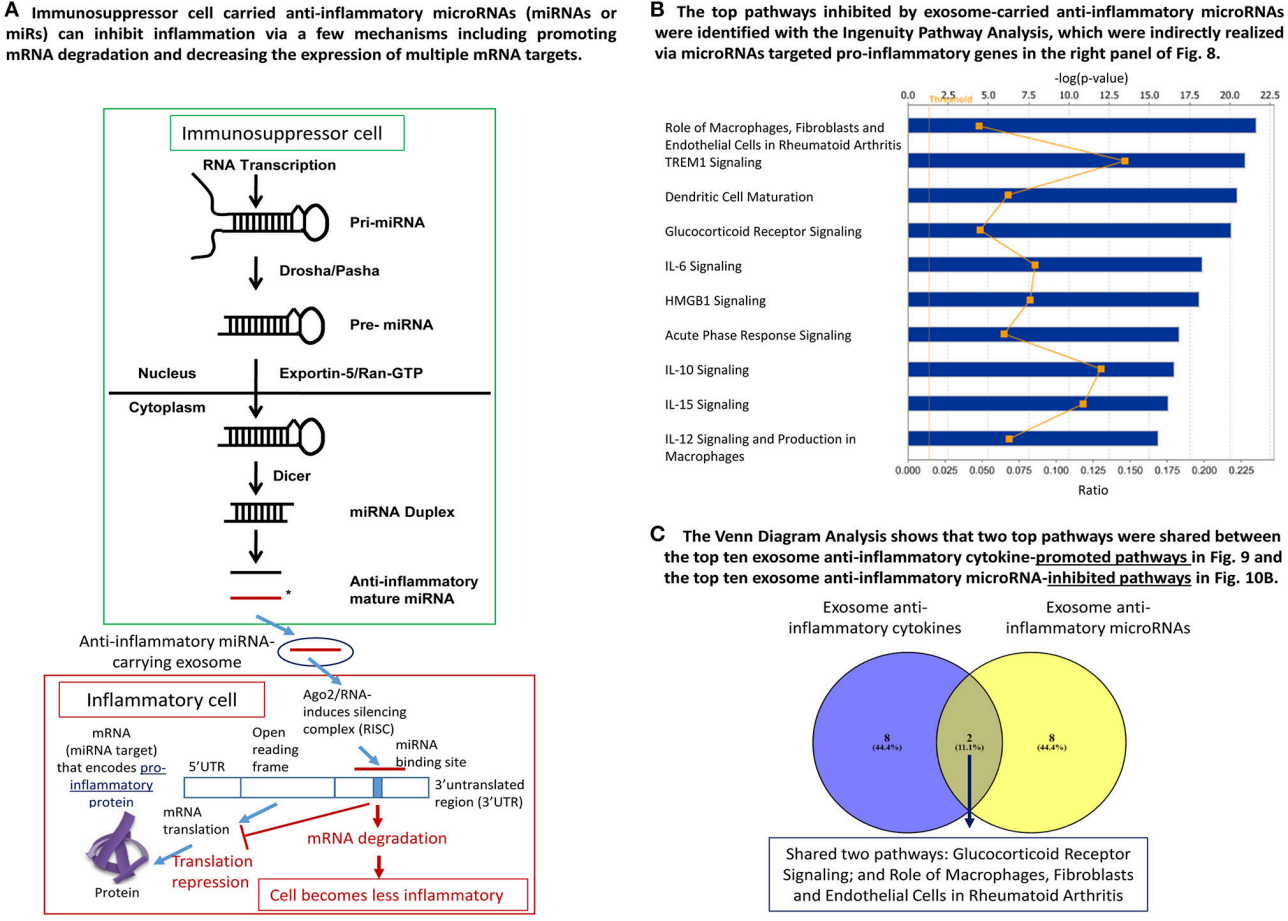
MiRNA in immune cell derived exosomes exert anti-inflammatory effects. **(A)** Graphical representation of how miRNA inhibit inflammation by promoting mRNA degradation and decreasing the expression of multiple mRNA targets. **(B)** The major pathways that are inhibited by the miRNA that are transported in exosomes derived from immune cells. **(C)** Venn analysis revealed that only 2 signaling pathways are shared by the anti-inflammatory cytokines and miRNA found in the exosomes generated by immune cells.

As we and others reported, anti-inflammatory miRs have two mechanisms underlying their biological functions: inhibiting the expression of pro-inflammatory mRNAs and suppressing the protein translation of pro-inflammatory mRNAs (Figure 10A). In order to determine whether miRs show pleiotropy and regulate many mRNAs, we searched the miR database (http://mirtarbase.mbc.nctu.edu.tw/php/search.php) with experimentally verified mRNA targets. The results showed that except miR-124, all other miRs have many mRNA targets (left most panel of Figure 8), which explained why we could not find shared pathways with anti-inflammatory cytokines/growth factors and miRs with the Cytoscape Pathway Network analysis and Ingenuity Pathway Analysis shown in Figures 9A,B, respectively.

To understand the snapshot of the mechanisms underlying the roles of 18 exosome anti-inflammatory miRs, we conducted a literature search in the PubMed database (two right most panels of Figure 8) and identified experimentally verified pro-inflammatory mediators that are downregulated by these miRs. We found that these miRs can induce significant effects on pro-inflammatory molecules including CD40 (T cell co-stimulating molecule), Toll-like receptor 4 (TLR4, a prototypic pathogen-associated molecular pattern receptor for bacterial endotoxin lipopolysaccharide) (Yang et al., 2008), IL-6, C-C motif chemokine ligand 2 (CCL2), IKB kinase α (IKKα), NF-kB, extracellular signal-regulated kinase 1/2 (ERK1/2)-mitogen activated protein kinase (MAPK), p38-MAPK (Mai et al., 2016), tumor necrosis factor-α (TNF-α) and IL-1β (Yin et al., 2013). To further understand how exosome-carried anti-inflammatory miRs inhibit inflammation via suppressing these sets of pro-inflammatory molecules, we performed the Ingenuity Pathway Analysis. As shown in Figure 10B, the exosome-carried anti-inflammatory miRs inhibit the inflammation of target cells at least via the top 10 pathways represented by the pro-inflammatory molecules in the right panel of Figure 8. The top 10 pathways included role of macrophages, fibroblasts, and endothelial cells in rheumatoid arthritis, triggering receptor expression on myeloid cells-1 (TREM1) signaling, dendritic cell maturation, glucocorticoid receptor signaling, IL-6 signaling, high mobility group box chromosomal protein 1 (HMGB1) signaling, acute phase response signaling, IL-10 signaling, IL-15 signaling, and IL-12 signaling and production in macrophages.

We then examined whether exosome-carried anti-inflammatory cytokines/growth factors and anti-inflammatory miRs share any pathways in inhibiting inflammation of target cells. We performed the Venn analysis and found that immunosuppressor cell-derived exosome-carried anti-inflammatory cytokines/growth factors and anti-inflammatory miRs share two pathways such as glucocorticoid receptor signaling and role of macrophages, fibroblasts and endothelial cells in rheumatoid arthritis (Figure 10C). Our results have demonstrated that immunosuppressor cell-derived exosome-carried anti-inflammatory cytokines/growth factors and anti-inflammatory miRs have multiple anti-inflammatory pathways to inhibit the inflammation of target cells.

## Discussion

**Figure 11:**
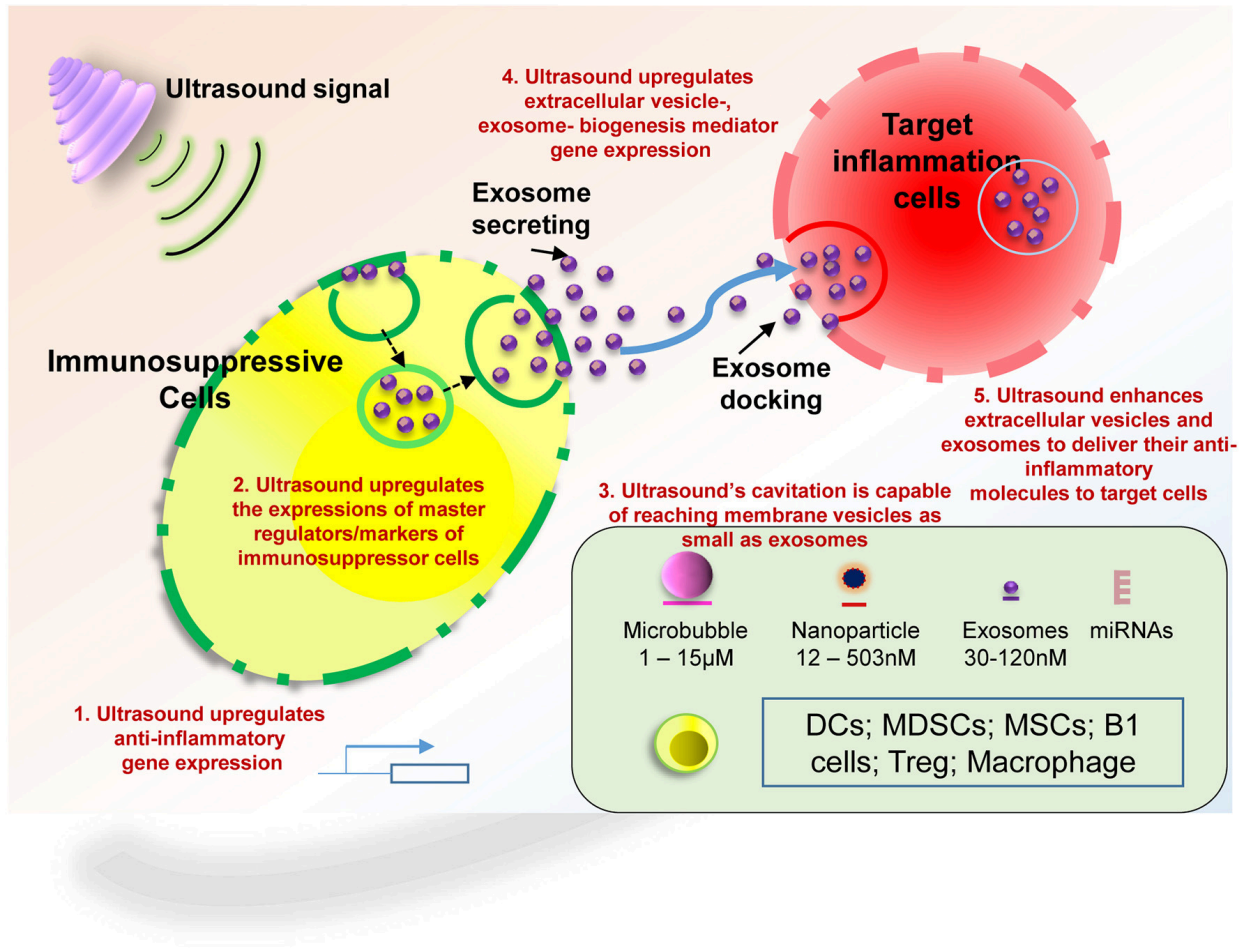
Our novel working model which proposes the potential anti-inflammatory mechanisms utilized by LIUS to exert its beneficial effects. These potential mechanisms are as follows; (1) LIUS upregulates the expression of anti-inflammatory/immunosuppressive genes in the ultrasound-treated cells; (2) LIUS enhances the expression of the master regulators (markers) of immunosuppressor cells; (3) LIUS mediated cavitation is capable of reaching the membrane vesicles as small the expression of extracellular vesicles; and (4) LIUS increases exosome biogenesis and docking mediators, and (5) Ultrasound enhances extracellular vesicles and exosomes to deliver their anti-inflammatory molecules to target cells. Our new findings suggest that LIUS inhibits inflammation via above discussed novel mechanisms to make the target inflammatory cells become less inflammatory. DCs, dendritic cells; MDSCs, myeloid-derived suppressor cells; MSCs, mesenchymal stem cells; B1 cells, CD5+ B1 B cells; Treg, CD4+Foxp3+ regulatory T cells.

Therapeutic applications of ultrasound in addition to its use in diagnosis have been accepted to be clinically beneficial. These benefits are the result of low intensity ultrasound (LIUS), which avoids the cell death and tissue damage associated with high intensity focused ultrasound (HIFU) that has been used as a surgical tool (Izadifar et al., 2017). As shown in Figure 2A, the anti-inflammatory effects are responsible for the clinical benefits of LIUS (Tabuchi et al., 2007; Hundt et al., 2008; Lu et al., 2009). However, the molecular mechanisms underlying the anti-inflammatory effects of LIUS remain poorly defined. Determination of the novel molecular mechanisms underlying the anti-inflammatory properties of LIUS would significantly improve our understanding on this important issue and allow for the development of LIUS-based therapeutics. To fill in this important knowledge gap, in this study, we used cutting-edged molecular database mining approaches we pioneered in 2004 (Ng et al., 2004; Yin et al., 2009; Li et al., 2012; Shao et al., 2016). Our data analysis revealed the following significant findings which we have also elaborated in Figure 11: (1) anti-inflammatory effects of LIUS are mediated by upregulation of anti-inflammatory gene expression; (2) LIUS upregulates the markers and master genes of immunosuppressor cells such as MDSCs, MSCs, B1-B cells and Treg; (3) LIUS not only can be used as novel therapeutic approaches to deliver drugs packed in various structures such as nanobeads, nanospheres, polymer microspheres, lipidosomes, but also can make use of natural membrane vesicles as small as exosomes derived from immunosuppressor cells as a novel mechanism to fulfill its anti-inflammatory effects; (4) LIUS induces the expression of extracellular vesicle/exosome biogenesis mediators and extracellular vesicle/exosome docking mediators; (5) The majority of top pathways are not shared between LIUS-induced anti-inflammatory genes and LIUS-induced immunosuppressor cell markers, suggesting that LIUS uses multiple cellular sensors-linked transcription pathways; and (6) anti-inflammatory cytokines and anti-inflammatory miRs in exosomes inhibit inflammation of target cells via multiple shared and specific pathways.

Sonoporation mediated membrane disruption and cavitation are important biological effects created by ultrasound together with microbubbles. Microbubble mediated cavitation relies on the magnitude of expansion and collapse during bubble oscillation, and also can generate significant physical effects such as light emissions, high pressure, fluid flow, shear stress, shockwaves and “micro jets” (Marmottant and Hilgenfeldt, 2003; Hwang et al., 2005; Ohl et al., 2006; Miller, 2007; Wu, 2007). Microbubble-assisted drug delivery has especially gained attention as a newly emerging therapy in recent years (Bao et al., 1997; Mehier-Humbert et al., 2005). Ultrasound–microbubble cavitation physically punctures the plasma membrane on a transient basis (Mehier-Humbert et al., 2005). Overstretching the cell membrane causes reparable submicron pore formation, providing primary evidence of low amplitude (0.12 MPa at 0.834 MHz) ultrasound sonoporation mechanism (Nejad et al., 2016). During such a process, the actin cytoskeleton may be disrupted in tandem because this network of subcellular filaments is physically interconnected with the plasma membrane (Schiffer et al., 2014). Sonoporation also can transiently disrupt cellular membranes and cause concomitant perturbation of actin cytoskeleton (Schiffer et al., 2014). In addition, ultrasound at low diagnostic power, causing stable oscillations of the microbubbles, results in a transient increase in membrane permeability for Ca2 (Juffermans et al., 2006; Park et al., 2010). Taken together, all these factors may profoundly affect cellular sensors that can activate various downstream signaling pathways in tissues exposed to LIUS.

The sensors present in the cells that are responsible for detecting the physical effects exerted by LIUS has not been extensively studied. It is highly likely that thermal effects exerted by LIUS may have the ability to change the conformation of proteins and thus alter their activity. The temperature dependent changes in certain proteins such as TRP (Transient Receptor Potential) ion channels itself may act as thermal sensors in cells (Sengupta and Garrity, 2013). Whether LIUS can specifically activate TRP ion channels has not been reported yet. Furthermore, the shockwaves created by LIUS may have the ability to activate the sensors that are responsible for mechano-sensation and transduction. Previously it was suggested that integrins found on the cell surface may act as mechanotransducers to transmit acoustic energy to the fibroblasts (Zhou et al., 2004; Cheng et al., 2014). Sensors that can detect shear stress produced by LIUS has not been reported yet. Fluid shear stress does not involve traditional receptor/ ligand binding, therefore, identifying the molecules that sense shear stress has been difficult (White and Frangos, 2007). It is suggested that cellular membranes itself may act as shear stress sensors (Katoh et al., 1995; Gudi et al., 1998). Activation of heterotrimeric G proteins has been reported in response to changes in shear stress in endothelial cells (Gudi et al., 1996). Nevertheless, further experiments are needed to identify the cellular sensors that capture LIUS mediated physical effects.

Activation of the cellular sensors by the physical properties exerted by LIUS may trigger many downstream signaling pathways. Previously it was shown that LIUS exposure could positively impact inflammatory process, improved collagen alignment and increased COX-2 (cyclo-oxygenase-2) expression in rat skeletal muscle exposed to cryolesion (Montalti et al., 2013). LIUS mediated anti-inflammatory effects were further validated by Nakao et al., who demonstrated that LIUS inhibits LPS mediated toxicity by attenuating TLR4 signaling pathway in murine osteoblasts (Nakao et al., 2014). Additionally, LIUS was reported to promote cell proliferation, thus wound healing by activating ERK1/2 via ROCK (Rho kinase/Rho-associated coiled-coil-containing protein kinase) dependent mechanism (Zhou et al., 2004). Furthermore, the ability of LIUS to modulate integrin and subsequently activate FAK (focal adhesion kinase)/PI3K (phosphatidylinositol 3-kinase)/AKT in chondroyctes was reported (Cheng et al., 2014). The same study revealed that LIUS mediated activation of integrin/ FAK/ PI3K/AKT pathway altered the extracellular matrix production by chondrocytes in osteoarthritis. In line with this evidence, inhibition of PI3K/AKT pathway significantly reduced the protective biological effects activated by LIUS in osteoarthritis (Zhang et al., 2016). Also, the angiogenic potential of LIUS was reported previously. LIUS treatment in porcine model with myocardial infarction resulted in increased capillary density in the ischemic region. Further analysis revealed that LIUS treatment significantly increased VEGF (vascular endothelial growth factor), bFGF (basic fibroblast growth factor) and eNOS (endothelial nitric oxide synthase) (Hanawa et al., 2014). Therefore, it is evident that LIUS can trigger molecular signaling cascade in the exposed tissues and stimulate various biological responses.

Our analysis revealed that ultrasound can significantly impact exosome biogenesis and docking. Exosomes are considered mediators of intercellular communication by the transfer of proteins and RNA (Zhang et al., 2014). Our results emphasize more on LIUS mediated physical effects such as heat-, shock wave- and shear stress-triggered signaling can induce extracellular vesicle/exosome biogenesis and extracellular vesicle/exosome docking than on LIUS cavitation-triggered membrane vesicle release of exosome contents. Taken together, our results suggest that both mechanisms can facilitate the immunosuppressor cell-derived exosome production and docking that mediate anti-inflammatory function.

Dendritic cell (DC)-derived exosomes have been characterized extensively at the ultrastructural and protein levels (Raposo et al., 1996; Clayton et al., 2001; Skokos et al., 2003). These results imply that DC-derived exosomes suppress inflammation and autoimmunity through a MHC class II-dependent pathway in an Ag-specific manner by modulating the activity of both endogenous T cells and APCs (Kim et al., 2005). Highly purified DC-derived exosomes have been shown to contain certain cytosolic proteins such as tubulin, actin, and certain actin-binding proteins, as well as MHC class I and II, T-cell-costimulatory molecules CD86, ICAM-1, lamp-2, the aM-h2 integrin, the tetraspanins CD9 and CD63, and MFGE8/ lactadherin (Raposo et al., 1996; Escola et al., 1998; Thery et al., 1999, 2001), suggesting that they play important roles in immune regulation (Ghivizzani et al., 2000). CD4+CD25+ Foxp3+ Treg cells control immune responses and maintain immunological tolerance (Sakaguchi et al., 1995). CD73-expressing exosomes produced by Treg cells following activation contribute to their suppressive activity through the production of adenosine (Smyth et al., 2013). MDSC exosomes were assessed for immunological properties such as cytokine induction in monocytes and the induction of Tregs through splenocytes or peripheral blood mononuclear cells (PBMCs) (English et al., 2009; Tasso et al., 2009). MSC exosomes are immunologically active, which have the potential to attenuate an activated immune system through the induction of anti-inflammatory cytokines and Tregs. Infusion of MDSC exosomes enhanced the survival of allogenic skin graft in mice and increased Tregs (Zhang et al., 2014). Therefore, exosomes are thought to play an important role in intercellular communication and are produced by many different cell types including CD4+ and CD8+ T cells (Blanchard et al., 2002; Busch et al., 2008; Xie et al., 2010; van der Vlist et al., 2012). However, whether LIUS anti-inflammatory effects use exosomes as a molecular mechanism remains unknown. Our new results toward addressing this question have demonstrated three significant findings: *first*, LIUS induces upregulation of immunosuppressor cell markers and master regulators, suggesting that LIUS promotes immunosuppressor cell generation; *second*, LIUS induces the upregulation of extracellular vesicle/exosome biogenesis mediators; and *third*, LIUS induces the upregulation of extracellular vesicle/exosome docking mediators. Further, the majority of the signaling pathways mediated by the main stream mechanisms we identified are not shared. Therefore, as we emphasized above, LIUS mediated physical effects such as heat (Palkar et al., 2015)-, shock wave (Pang et al., 2016)-, and shear stress-sensors (Kalapesi et al., 2005) might be sensed by multiple but distinct sensors that trigger various downstream transcription factors and signaling pathways.

## Conclusions

There is a significant knowledge gap in understanding the molecular mechanisms involved in LIUS mediated anti-inflammatory effects. Herein, we provide novel insights in to potential anti-inflammatory mechanisms that may be utilized by LIUS. Nevertheless, we acknowledge that carefully designed *in vitro* and *in vivo* experimental models are needed to further verify the LIUS mediated anti-inflammatory mechanisms we report here. These experimental models will enable to consolidate the efficacy of LIUS as a therapy in various pathological conditions as well. However, our analysis provides a stepping stone to better understand the potential mechanisms mediated by LIUS to exert its beneficial anti-inflammatory effects. Also, our findings provide molecular readouts that can be used to determine optimal ultrasound intensity and duration and will provide guidance for the development of the future LIUS therapeutics for cancers, inflammation, tissue regeneration, and tissue repair.

## Author contributions

QY carried out the data gathering, data analysis and prepared tables and figures. GN, CD, YuS, CJ, RC, HF, YS, LW, WY, PT, LL, SG, XZ, MK, and HW aided with analysis of the data. XY supervised the experimental design, data analysis, and manuscript writing. All authors read and approved the final manuscript.

### Conflict of interest statement

The authors declare that the research was conducted in the absence of any commercial or financial relationships that could be construed as a potential conflict of interest.
